# The role of neutrophil death in chronic inflammation and cancer

**DOI:** 10.1038/s41420-020-0255-6

**Published:** 2020-04-22

**Authors:** Christine Brostjan, Rudolf Oehler

**Affiliations:** Department of Surgery, Medical University of Vienna, Vienna, Austria

**Keywords:** Cancer microenvironment, Immune cell death, Granulocytes, Cell death and immune response

## Abstract

The lifespan of a neutrophil is short and limited by programmed cell death, followed by efferocytosis. When activated or exposed to insult, neutrophil death may be delayed to support neutrophil effector functions such as phagocytosis, cytokine release, and pathogen destruction by degranulation. However, neutrophils may also alter the type of cell death and thereby affect inflammatory responses and tissue remodeling. This review briefly introduces the various forms of neutrophil death including apoptosis, necrosis/necroptosis, and the formation of so-called “neutrophil extracellular traps” (NETs), and it summarizes the clearance of dead cells by efferocytosis. Importantly, distinct types of neutrophil death have been found to drive chronic inflammatory disorders and cancer. Thus, the tumor and its microenvironment can delay neutrophil apoptosis to exploit their pro-angiogenic and pro-metastatic properties. Conversely, neutrophils may enter rapid and suicidal cell death by forming extracellular traps, which are expelled DNA strands with neutrophil proteins. Components of these DNA–protein complexes such as histones, high-mobility group protein B1, or neutrophil elastase have been found to promote cancer cell proliferation, adhesion, migration, invasion, and thereby tumor metastasis. In other settings of chronic inflammatory disease such as gout, NETs have been found protective rather than detrimental, as they promoted the local degradation of pro-inflammatory cytokines by neutrophil proteases. Thus, the interaction of neutrophils with the tissue environment extends beyond the stage of the living cell and the type of neutrophil death shapes immune responses and tissue remodeling in health and disease.

## Neutrophil life cycle

**Fig. 1:**
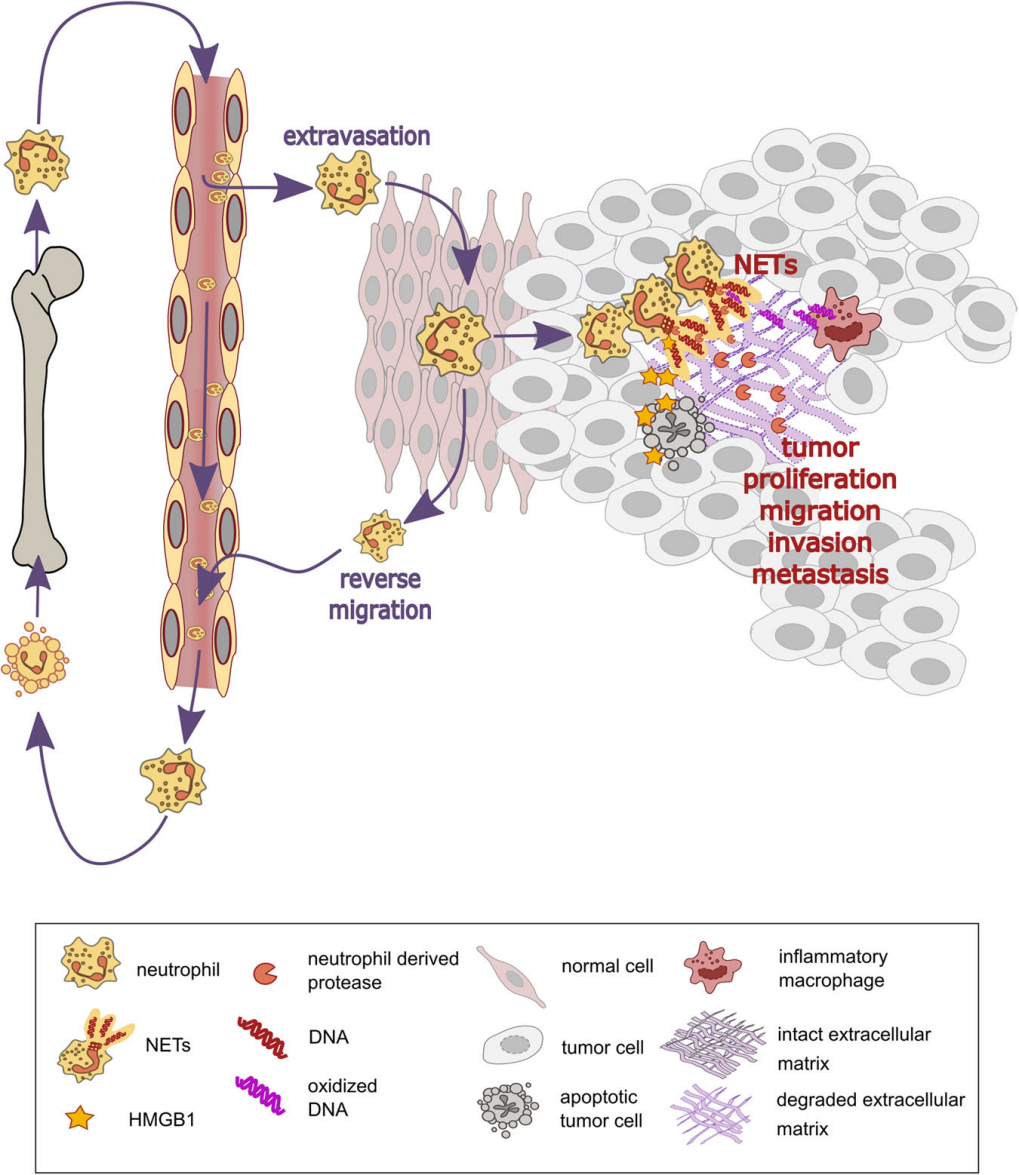
Neutrophil extracellular traps in cancer. Neutrophils originating from bone marrow have a short lifespan in circulation, which is controlled by programmed cell death. When attracted by chemokines, they extravasate into tumor tissue where they are activated to delay apoptosis and engage in the inflammatory tumor microenvironment. A fraction of activated neutrophils may reverse migrate and home back to the bone marrow which shapes further neutrophil release. The tumor-invading neutrophils are exposed to hypoxia as well as cancer and stroma cell signals, which can trigger the formation of neutrophil extracellular traps (NETs). NET components such as oxidized DNA may stimulate an inflammatory response by macrophages or dendritic cells. NET-associated proteases alter the extracellular matrix and NET-derived HMGB1 molecules activate cancer cells to jointly promote tumor cell proliferation, migration, invasion, and metastasis.

Neutrophils provide the first line of defense against invading pathogens. Under normal conditions they are produced at numbers of 10^11^ per day and survive only a few hours to days in circulation^1,2^. In case of infection or tissue damage neutrophils migrate to the affected site in response to chemoattractants, such as CXCL8 (IL-8). During transmigration through the endothelium VCAM-1 on the inflamed endothelial cells interacts with integrin α_9_β_1_ on neutrophils. This stimulates the release of GM-CSF, which increases their life time by an auto-endocrine loop^3^. Delaying apoptosis is an important mechanism for neutrophil accumulation at sites of inflammation. When activated by pathogen- or damage-associated molecular patterns (PAMPs or DAMPs) neutrophils contribute to the elimination of pathogens by phagocytosis, degranulation, release of ROS, and formation of so-called neutrophil extracellular traps (NETs)^4^. Furthermore, neutrophils recruit and activate additional leukocytes by the release of pro-inflammatory mediators and promote tissue remodeling while preventing pathogen spread. Exhausted neutrophils are then either removed locally by phagocytes or remain as a major constituent of pus. In addition, they can also reverse migrate via the blood stream to the bone marrow, where they undergo apoptosis (Fig. 1, left)^5,6^. The number of circulating neutrophils is tightly regulated. They are cleared in the bone marrow, spleen, and liver. Bone marrow macrophages release G-CSF in response to uptake of returning neutrophils, which then stimulates the release of new neutrophils into the blood flow^7^. The essential contribution of living neutrophils and their subtypes in cancer and other diseases have been described in numerous reviews elsewhere^8–10^. Here, we focus on neutrophil cell death and the fundamental impact of its deregulation in chronic disease and cancer.

### Ways of neutrophilic cell death

There is a considerable variety of different ways for neutrophils to die which entail distinct consequences in health and disease. To date apoptosis, pyroptosis, necrosis, necroptosis, autophagic cell death, and NETosis have been described^11^. The survival time of mature neutrophils is subject to regulation by both, the intrinsic and the extrinsic pathway of apoptosis. Although neutrophils host only few mitochondria they have a functional cytochrome c/caspase 3-mediated cell death pathway^12^. However, their balance of pro- and anti-apoptotic members of the Bcl-2 superfamily differs from other cell types^13^. Mature neutrophils exhibit low expression of Bcl-2 but high levels of Mcl-1^12^. The data on the expression of Bcl-xL protein are controversial. With respect to the extrinsic pathway, interactions of the surface molecules Fas/FasL and TRAIL/TRAILR can induce apoptosis as in other cell types. In contrast, TNFα-induced cell death seems to differ strikingly in neutrophils. TNFα induces, delays or has no effect on neutrophil apoptosis in dependence on its concentration^12^. If apoptotic neutrophils are not removed in time, they progress toward secondary necrosis. This is associated with a passive release of caspase 3-processed IL-6C tetramers and MIF oligomers, which are considered to act as danger signals^14^. Neutrophil apoptosis can be also initiated by phagocytosis via the cell surface molecule Mac-1 and subsequent caspase 8/3 activation^15^. Of interest, some pathogens interfere with phagocytosis-induced cell death and extend the lifespan of neutrophils to promote their own replication within the cells^16^. A mechanism to prevent such a replication is pyroptosis. It is defined by the activation of caspase 1 or caspases 4/5/11 instead of caspase 3 and involves NLRC4 or NLRP3/ASC inflammasome activation^17^. Pyroptosis has mainly been observed when neutrophils cannot respond to bacterial infection via ROS formation by NADPH oxidase^18^.

Neutrophil death by necrosis is usually a trigger for inflammation^19^. It may occur in a regulated fashion. This necroptosis is coordinated by RIPK1, RIPK3, and MLKL, which finally results in a compromised membrane integrity and the release of cytosolic components^20^. Necroptosis can be triggered by engagement of CD44, CD11b, CD18, or CD15 on GM-CSF-primed neutrophils^21^ or by autophagy^22^.

Another option of neutrophil cell death was discovered in 2004 when NETs were first described as expelled DNA strands decorated with neutrophil proteins and intended to entrap and eliminate pathogens^23^. It was found that the decondensed DNA is suited to entangle microorganisms and that associated histones are highly toxic. Furthermore, neutrophilic enzymes such as MPO or elastase contribute to pathogen destruction^24,25^. Although the formation of NETs was originally described as a particular type of neutrophil cell death and hence termed NETosis, it was subsequently found that NETs may also be generated without immediate cell death^26^.

Suicidal NETosis centrally involves the decondensation of nuclear DNA. Depending on the stimulus, this process may be mediated by ROS production of NADPH oxidase and subsequent intracellular release of MPO and elastase from neutrophil granules. The nuclear translocation of these enzymes allows for histone cleavage, chromatin decondensation, and further pore formation in granule as well as cytosolic membranes^27^. Although this pathway is triggered by, e.g., *Aspergillus nidulans*^28^, other pathogens such as *Pseudomonas aeruginosa* seem to initiate alternative mechanisms of NET formation^29^. Chromatin decondensation may be facilitated by the enzyme peptidylarginine deiminase 4 (PAD4), which mediates histone citrullination^30^ and has been shown to contribute to the antibacterial defense against *Shigella flexneri* and group A *Streptococcus pyogenes*^30^. Importantly, in addition to nuclear DNA, neutrophils may expel mitochondrial DNA (by suicidal or vital NETosis). As the mitochondrial DNA is less protected by complexed proteins, it is highly oxidized during the process and constitutes a major pro-inflammatory trigger when released during NET formation^31–33^.

### Clearance of apoptotic neutrophils

Apoptotic cells can be removed by various categories of phagocytes via efferocytosis. It represents a version of stimulated micropinocytosis and is distinct from the classical phagocytosis of microbes^7^. The most prominent eat-me signal on the surface of apoptotic cells is phosphatidylserine, which is recognized by different receptors in the phagocytic synapse. Some of them bind directly to phosphatidylserine (SIRPα, TIM4, and BAI1) while others (MERTK and AXL receptor tyrosine kinases, complement receptors, and integrins) require bridging factors such as GAS6, protein S, C1q, C3, or MFGE8. The involved signal pathways have been described extensively elsewhere^34,35^. Apoptotic neutrophils upregulate annexin-I and calreticulin on their surface which act as supplementary eat-me signals^36^. Blockade of a single receptor has never been shown to completely abolish efferocytosis, suggesting that either redundant efferocytosis pathways exist or individual receptors cooperate with other receptors.

Efferocytosis activates an inflammosuppressive and immunosuppressive response in the phagocyte^37^. Binding of phosphatidylserine to MERTK and AXL blocks TLR and type 1 IFN pathways, whereas binding to TIM1 inhibits the secretion of TNFα, IL-6, and CCL5. Upon engulfment of dying cells, LC3 is recruited to the dead cell-containing phagosome^35^. LC3-decorated phagosomes promote the production of IL-10 and TGFβ. The anti-inflammatory response relies on a prolonged presence of apoptotic cells^36^. A short exposure has no effect. Interestingly, efferocytosis of neutrophils with surface exposed granule protein PR3 promotes a pro-inflammatory rather than anti-inflammatory response^38^. Efferocytosis has been shown not only to contribute to the resolution of inflammation but also to promote the proliferative and remodeling phases of tissue repair (reviewed in ref. ^39^). It activates the synthesis of lipoxins, DHA products, and E series resolvins and decreases their production of classical eicosanoids^39,40^. These pro-resolving lipid mediators collectively reduce vascular permeability, inhibit further neutrophil transmigration, promote recruitment of non-phlogistic monocytes, induce neutrophil apoptosis and promote their efferocytosis, creating a positive feedback in favor of resolution.

It is well accepted that also neutrophils themselves have the capacity to efferocytose apoptotic cells but there is very little literature available. This ability depends on bridging factors and increases after activation of neutrophils with GM-CSF, TNFα, IFNγ, or TLR agonists^41,42^. After efferocytosis neutrophils block respiratory burst and reduce the release of pro-inflammatory TNFα and increase the secretion of CXCL8^41^. In response to tissue injury, local neutrophils initiate a highly coordinated form of chemotaxis of further neutrophils involving a sequence of auto- and paracrine signaling of chemokines, lipids, and chemoattractants^43^. This “neutrophil swarming” leads to an accumulation of a high number of neutrophils in the damaged tissue, which usually exceeds by far the number of macrophages. This suggests that efferocytosis of apoptotic cell debris by neutrophils is a frequent event in an inflamed tissue or in a tumor microenvironment and may contribute to a considerable degree to the local resolution of inflammation und tissue regeneration.

### Neutrophil cell death in disease

#### Apoptosis

**Fig. 2:**
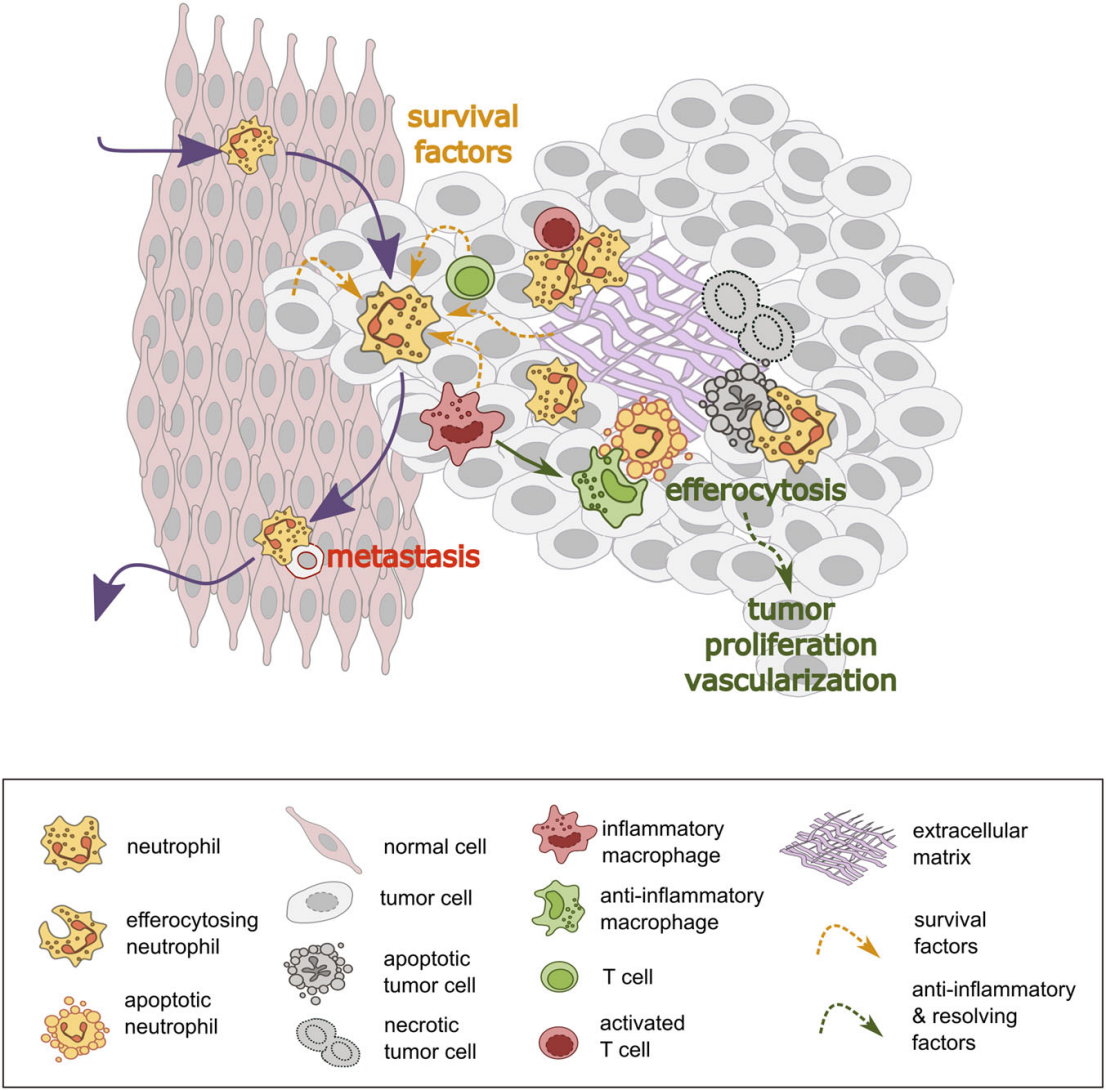
Neutrophil apoptosis and efferocytosis in cancer. Tissue-infiltrating neutrophils that are attracted by tumor-derived signals are exposed to a variety of survival factors originating from tumor cells, stroma, hypoxia, or dying cells. They may propagate the inflammatory tumor microenvironment by recruiting and activating further leukocytes, such as cytotoxic T-cells. Tumor-associated neutrophils have the potential to reverse migrate into circulation, thereby facilitating metastasis of attached tumor cells. However, the majority of them is proposed to undergo local apoptosis and subsequent efferocytosis by macrophages, which drives an anti-inflammatory M2-like polarization, tumor proliferation, and vascularization. Conversely, neutrophils may remove apoptotic tumor cells by efferocytosis and thereby promote tissue remodeling and cancer growth.

Deregulated neutrophil apoptosis is often linked to disease. An increased rate has been reported in different neurodegenerative disorders^44^. In contrast, autoimmune diseases and cancer are frequently associated with reduced neutrophil apoptosis^45^. Many solid tumors including colorectal cancer, lung cancer and breast cancer are characterized by a high neutrophil infiltration^46–48^. However, its predictive value differs between cancer types^8^. Tumor-associated neutrophils show a prolonged lifespan (Fig. 2). Numerous different survival factors for neutrophils have been described, including cytokines, chemokines, hormones, lipid mediators, and DAMPs (summarized in ref. ^3^). G-CSF for example, enhances the expression of PCNA in neutrophils which prevents apoptosis by sequestering pro-apoptotic caspases. Many cancer types secrete multiple neutrophil survival factors including G-CSF and IFNβ^49^. An additional contribution to neutrophil survival comes from GM-CSF and IFNγ from stromal macrophages, NK cells, and T cells^3,50^. Furthermore, DAMPs released from dying cells in response to tumor-associated tissue damage are able to prolong neutrophil lifespan^3^. Finally, also physicochemical conditions in the tumor microenvironment can promote neutrophil survival. For example, hypoxia activates an oxygen-sensing prolyl hydroxylase 3 in neutrophils, which mediates an increase of anti-apoptotic Bcl-x_L_^51^.

Although the final fate of tumor-associated neutrophils when they reach the end of their life time is not well characterized it is generally assumed that the majority of tumor-associated neutrophils undergo local apoptosis. It has been proposed that efferocytosis in the tumor microenvironment mediates an M2-like polarization of tumor-associated macrophages and that the related anti-inflammatory and pro-resolving response contributes to tumor growth and vascularization^52^ (Fig. 2). Furthermore, recent studies in breast cancer and melanoma models revealed that a part of tumor-associated neutrophils migrate reversely into the blood flow and are finally cleared in the bone marrow. Some of them aggregate with cancer cells before leaving the tumor microenvironment and promote their spreading^53,54^.

#### NETs

Although apoptotic cell death allows for the controlled removal of cells in an immunosuppressive manner, necrosis/necroptosis, and in particular, NETosis result in leakage of pro-inflammatory and toxic components into the extracellular space. Apart from the beneficial impact of NETs in combating infections, their detrimental role in the pathophysiology of many non-infectious diseases has been the focus of research in recent years^55^. The very same components that confer pathogen defense are also found toxic to the host environment. Although neutrophil-derived peroxidases and proteases contribute to extracellular matrix destruction, histones seem to be another prime culprit of damage^56^. Externalized histone H4 was found to propagate cell death and inflammation by inducing lysis of tissue cells.

Thus, NETs have been detected in numerous chronic inflammatory diseases and reported to substantially contribute to pathogenesis. In respiratory disorders such as cystic fibrosis and chronic obstructive pulmonary disease, NETs were found to block airways, contribute to fibrotic regions that foster bacterial replication and convey stimulatory signals to surrounding macrophages^57,58^. With respect to vascular disorders, a promoting function of NETs in atherosclerosis has been reported based on the analysis of ApoE-deficient mice lacking the neutrophil proteases elastase and PR3^59^. The loss of NETs in cholesterol-rich areas was associated with a threefold decrease in atherosclerotic lesions. With respect to the mechanism, cholesterol crystals were shown to trigger NETosis, which then promoted the activation of macrophages. NETs are thus participating in the inflammatory process of atherosclerosis, which may ultimately lead to arterial thrombotic events. However, NETs are also directly involved in thrombosis. The interaction of activated platelets with neutrophils at the site of plaque rupture is believed to trigger NETosis and the accumulation of active tissue factor on NETs^60,61^. Furthermore, NETs seem to provide a scaffold for platelet, erythrocyte, and fibrin deposition, and NET-exposed histones as well as neutrophil proteases such as elastase and cathepsin G are known to further promote platelet activation and to degrade inhibitors of coagulation^62^. In particular, extracellular histones were found to activate platelets via TLR2 and TLR4, thereby inducing a procoagulant platelet phenotype^63^. Comparable effects have been proposed for NETs in venous thrombosis where NETs were further described to bind and activate factor XII in thrombogenesis^64,65^.

In addition to cholesterol, crystals, also urate crystals, in gout patients have been reported to trigger NET formation. Of particular interest, NETs were found to form protective aggregates (so-called “aggregated NETs” or “aggNETs”) in this setting that promoted the local degradation of pro-inflammatory cytokines by neutrophil proteases, thereby alleviating rather than aggravating disease symptoms^66^. The concept of beneficial versus detrimental forms of NETs was further extended to other types of non-infectious disease^67,68^. Therefore, it seems of importance to carefully characterize the mechanisms of NET formation associated with distinct disorders.

In line, it has recently been revealed that oxidized mitochondrial DNA rather than nuclear DNA expelled during NETosis drives damaging inflammatory reactions via dendritic cell activation and release of interferon alpha in patients with systemic lupus erythematosus (SLE)^69,70^. These patients were shown to accumulate a population of low-density granulocytes with an enhanced capacity for mitochondrial ROS production and mitochondrial NET formation. As a trigger for NETosis, autoreactive antibodies were identified. When mitochondrial ROS production was blocked in a mouse model of SLE, disease symptoms were significantly reduced^31,70^.

In the cancer setting, NETs were shown to promote metastasis, support the survival of tumor cells in circulation and even stimulate tumor invasion^71,72^. Specifically, cancer cells as well as intratumoral hypoxia were identified as inducers of NET formation, and NET-associated HMGB1 was found to activate cancer cells to promote their adhesion, proliferation, migration, and invasion^73^ (Fig. 1). Moreover, NETs and intact neutrophils can “catch” tumor cells via Mac-1/ICAM-1 interaction and thereby facilitate their adhesion for metastasis^74^. More recently, it was reported that NETs may also “awaken” dormant cancer cells. NETs induced by a pro-inflammatory trigger mediated the proteolytic remodeling of the matrix component laminin to reveal a novel epitope that triggered proliferation of dormant cancer cells via integrin activation^75^.

### Conclusion

All the data listed above confirm that apart from the role of active neutrophils in health and disease their influence on immune reactions and chronic disorders extends to the stage of neutrophil death. Although programmed cell death controls the lifespan and non-immunogenic clearance of cells, deregulated neutrophil apoptosis promotes chronic diseases like cancer by supporting stroma remodeling and metastasis. Alternatively, neutrophils may undergo a sudden suicidal form of cell death termed NETosis where expelled DNA–protein structures are highly toxic and proteolytic. In addition to their function in pathogen defense, NETs have been found in chronic inflammatory disorders. In particular, matrix remodeling, tumor growth, and metastasis are supported by components of this specific form of neutrophil death.

Neutrophils have become a prime target for medical intervention and an impressive number of clinical trials have been initiated in the last years (Table 1). Most studies focus on chronic diseases. However, their therapeutic approaches differ strongly from each other. Some studies apply neutrophil inhibitory strategies. Others try to inhibit neutrophil cell death. For example, chemotherapy-induced neutropenia is usually prevented by recombinant GM-CSF. It inhibits neutrophil apoptosis and stimulates neutrophil recruitment. Similar anti-apoptotic effects have been described for corticosteroids such as fluticasone or prednisolone. In contrast, antagonists of CXCR2 or depletion of IL-17 reduce neutrophil recruitment and NET formation. Blocking the IL-6 receptor with the antibody tocilizumab reduces the number of circulating neutrophils by an increased margination into the bone marrow. Also colchicine has inhibitory effects on neutrophils. It prevents the activation of the inflammasome and reduces neutrophil adhesion and recruitment. The increasing numbers of clinical studies confirm the central role of living and dying neutrophils in the various physiological and pathological conditions. The divergent therapeutic approaches that are applied reflect the fact that their contribution to the pathophysiology is highly disease-specific.

**Table 1:** Clinical trials aiming to modulate neutrophil function or survival.

Treatment	Outcome parameter	Condition	Phases	NCT number
Anti-IL-6R antibody (tocilizumab)	Neutrophil function, apoptosis and equilibrium	Healthy	Phase 4	NCT01991990
Anti-IL-6R antibody (tocilizumab)	Neutrophil apoptosis and activation	Rheumatoid arthritis	Phase 4	NCT01195272
Anti-IL-17 antibody (secukinumab)	Neutrophil function (phagocytosis), apoptosis, activation	Psoriatic arthritis	Phase 2	NCT02854163
Anti-phospholipid antibody	NETs formation and neutrophil function	Pregnancy loss	Not applicable	NCT03735108
Corticosteroid (fluticasone propionate)	Neutrophil count	Respiratory disease	Phase 1	NCT00869596
Corticosteroid (fluticasone propionate)	Neutrophil count	Respiratory disease	Phase 1	NCT01364519
Corticosteroid (prednisolone)	Neutrophil activation	Respiratory disease	Not applicable	NCT00159354
CXCR2 antagonist (AZD5069)	Neutrophil function (phagocytosis and oxidative burst)	Healthy	Phase 1	NCT01480739
CXCR2 antagonist (AZD5069)	Neutrophil count in bronchial biopsies	Respiratory disease	Phase 1	NCT01890148
CXCR2 antogonist (danirixin)	NETs formation and neutrophil function	Respiratory disease	Phase 2	NCT03250689
Dietary supplement: alcohol, caffeine	Neutrophil migration	Healthy	Not applicable	NCT02411318
Elastase antagonists (alvelestat)	Neutrophil function (elastase activity)	Graft vs. host disease	Phase 2	NCT02669251
Elastase antagonists (AZD9668)	NETs formation and neutrophil activation	Diabetes	Phase 2	NCT02597101
Hyperbaric oxygen	Neutrophil function (oxidative burst)	Infection	Phase 1	NCT02563678
Inflammasome disruption (colchicine)	Neutrophil activation	Cardiovascular disease	Not applicable	NCT03874338
P38 α MAPK inhibitors (PF03715455, PH797804)	Neutrophil count	Respiratory disease	Phase 1	NCT01314885
Pioglitazone, simvastatin, ibuprofen	Neutrophil count in oral mucosa	Cystic fibrosis	Not applicable	NCT00531882
rGM-CSF (sargramostim)	Neutrophil function (phagocytosis)	Infection	Phase 2	NCT01653665
Rifaximin-α (antibiotic)	Neutrophil function (spontaneous oxidative burst)	Liver cirrhosis	Phase 4	NCT02019784
